# A proposed radiological classification system of Hoffa’s fracture based on fracture configuration and consequent optimal treatment strategy along with the review of literature

**DOI:** 10.1051/sicotj/2019016

**Published:** 2019-06-07

**Authors:** Vaibhav Bagaria, Gaurav Sharma, Chaitanya Waghchoure, Rajendra M Chandak, Amit Nemade, Kalyan Tadepelli, Prashant Pawar

**Affiliations:** 1 Consultant Arthroplasty and Trauma Surgeon, Sir HN Reliance Foundation Hospital and Research Centre, Prarthana Samaj Girgaon 400004 Mumbai India; 2 Department of Orthopaedics, Sir HN Reliance Foundation Hospital and Research Centre, Prarthana Samaj Girgaon 400004 Mumbai India; 3 Chandak Nursing Home Nagpur 440012 Maharashtra India; 4 ORIGYN Clinic Nagpur 440010 Maharashtra India

**Keywords:** Hoffa fracture, Classification, Coronal fracture, Distal femur fracture

## Abstract

*Background*: Coronal fractures of distal end femur, referred as Hoffa’s fracture are not uncommon, yet easily missed injuries lacking proper classification system and consensus for ideal treatment. While most trauma surgeons adopt different strategies based on the fracture configuration and their own experience, there are no set ways to classify these based on the most appropriate treatment strategy. *Methods*: Thirty cases of Hoffa fracture from tertiary care centres were studied for the fracture pattern, fragment size, comminution and possible variations to formulate a radiological classification and treatment guidelines. Additionally, a literature search was used to analyze 77 case studies based on Hoffa fracture to find out the common fracture patterns and treatment modalities adopted for varying fracture patterns in these studies. Six independent observers participated in testing the inter-observer reliability of the proposed classification. *Results*: A new proposed radiological classification for Hoffa fracture consists of four main types. Type 1 is with fracture fragment >2.5 cm, Type 2 with fragment <2.5 cm, Type 3 is comminuted fracture, Type 4 are subdivided as Type 4a – Anterior, Type 4b – Bicondylar, Type 4c – Osteochondral type and Type 4d – With supracondylar extension. Optimum treatment modality depends on the type of Hoffa’s fracture and has been suggested in the study. Interobserver reliability demonstrated that overall agreement was 0.907692 with a fixed marginal Kappa of 0.881067 and free Marginal Kappa at 0.892308. Intra-observer reliability test for the classification system showed a strong Kappa value of +1.0. *Conclusion*: The new suggested classification helps identify different types of Hoffa’s fracture. This is likely to help decide optimal surgical treatment depending on the nature of the injury. The classification system has high inter and intra-observer reliability that enables its universal applicability.

## Introduction

Hoffa’s fracture is an eponymous term to denote the intra-articular fracture of distal end of the femur in coronal plane accounting for 0.65% of all the femur fractures [1, 2]. The first-ever description of this fracture dates back to 1869 (and not 1904 as it is known) when German surgeon Friedrich Busch mentioned it in an anatomical specimen from the knee joint of a cadaver [3]. The drawings of Busch were later used by Albert Hoffa in 1888 in the first edition of his textbook following which the fracture was recognized by his name [4].

Due to the intra-articular nature of this fracture, conservative management often possesses a risk of malunion, nonunion, knee instability and post-traumatic arthritis leading to compromised knee function [1, 2, 4, 5]. Therefore, current recommendations favour anatomical reduction and rigid internal fixation by a lag screw or a plate depending upon the fracture geometry and surgeon expertise [4, 6, 7]. Despite the agreement on operative management, dilemma still exists with respect to the fixation method, surgical approach, and prognosis, unlike many other fractures.

A classification system is used to describe various orthopaedic conditions and fractures. An ideal classification system is an enabler, it should be easy to use, provide an accurate description to enable communication between surgeons, aid in evaluating fracture preoperatively and also help in prognostication of the condition. Statistically, it should have good inter and intraobserver reliability and validity. The standardized AO–OTA classification describes these injuries under 33-B3 [8]. However, this classification does not further sub-classify different fracture patterns associated with Hoffa’s. The Letenneur classification, on the other hand, describes the mechanism of injury, but fails to offer the management in these injuries [4].

The aim of the present study was to classify various types of Hoffa’s fracture in a manner that is easy to reproduce and at the same time gives an insight into how to manage and prognosticate them along with rationale for adopting a particular management line on the basis of a series of 30 cases along with review of literature.

## Materials and methods

### Study cases

Thirty cases of Hoffa’s fracture were retrospectively reviewed at tertiary care trauma centre between 2013 and 2017. CT scans and radiographs of each case were studied to classify them into four broad types (Type I–IV) based on fracture pattern (primary coronal fracture line), fragment size, comminution, and other possible variations. Type IV was sub-classified into four subclasses.

### Literature search

**Figure 1:**
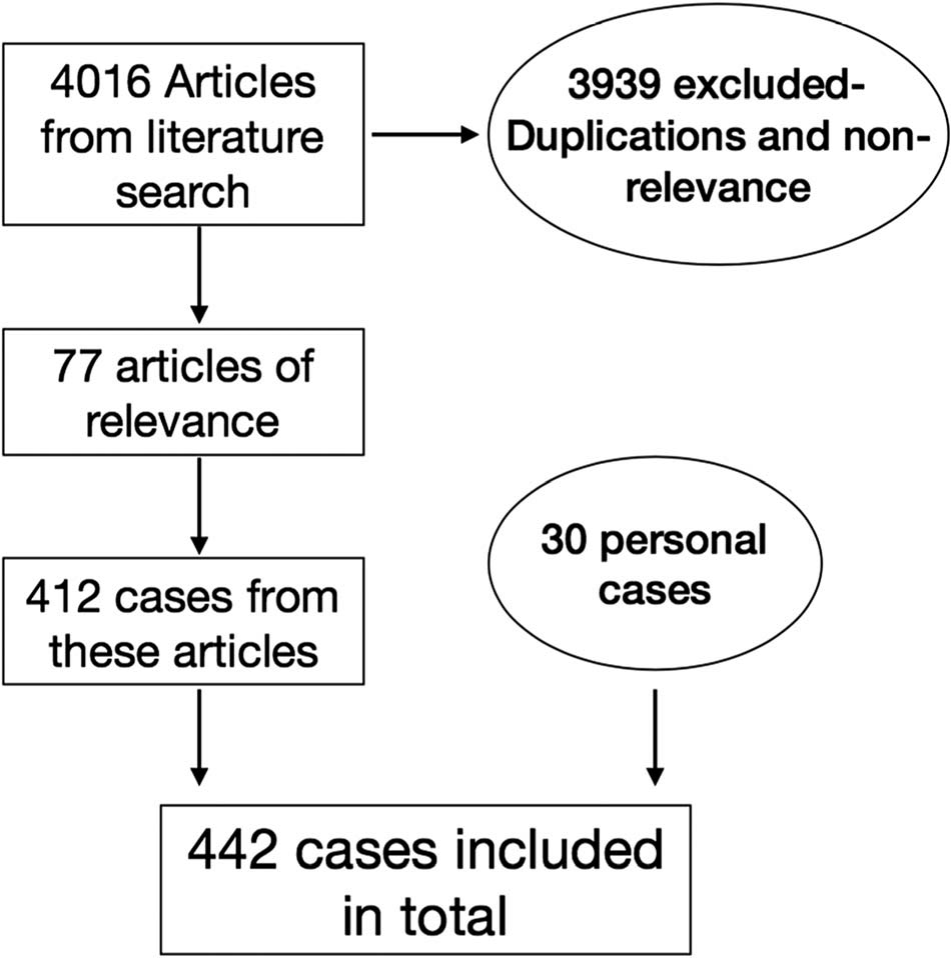
Flow chart to selection process.

A comprehensive literature search was performed using the terms: “Hoffa’s fracture”, “Coronal fracture”, “Osteochondral fracture distal femur”, “Letenneur classification”, “33B3”. Pubmed and Google Scholar were used as the primary search tools. English, as well as non-English articles (translated when required), were searched. Out of the 4016 articles, 77 were included in the study comprising a total of 412 cases (Figure 1). These cases were studied for the fracture patterns (if mentioned), surgical approaches and fixation devices used.

**Figure 2:**
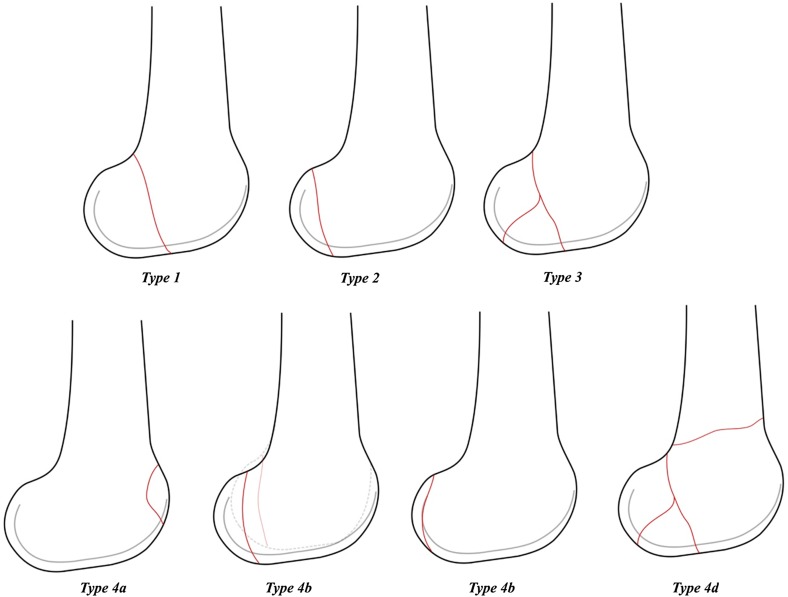
Sagittal View of various fracture configurations of Hoffa’s fracture as per the proposed classification system.

**Figure 3:**
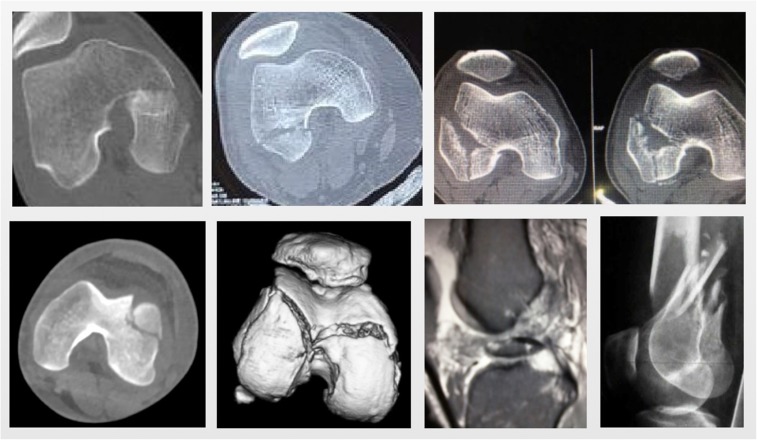
Radiological images as per the new classification system.

**Figure 4:**
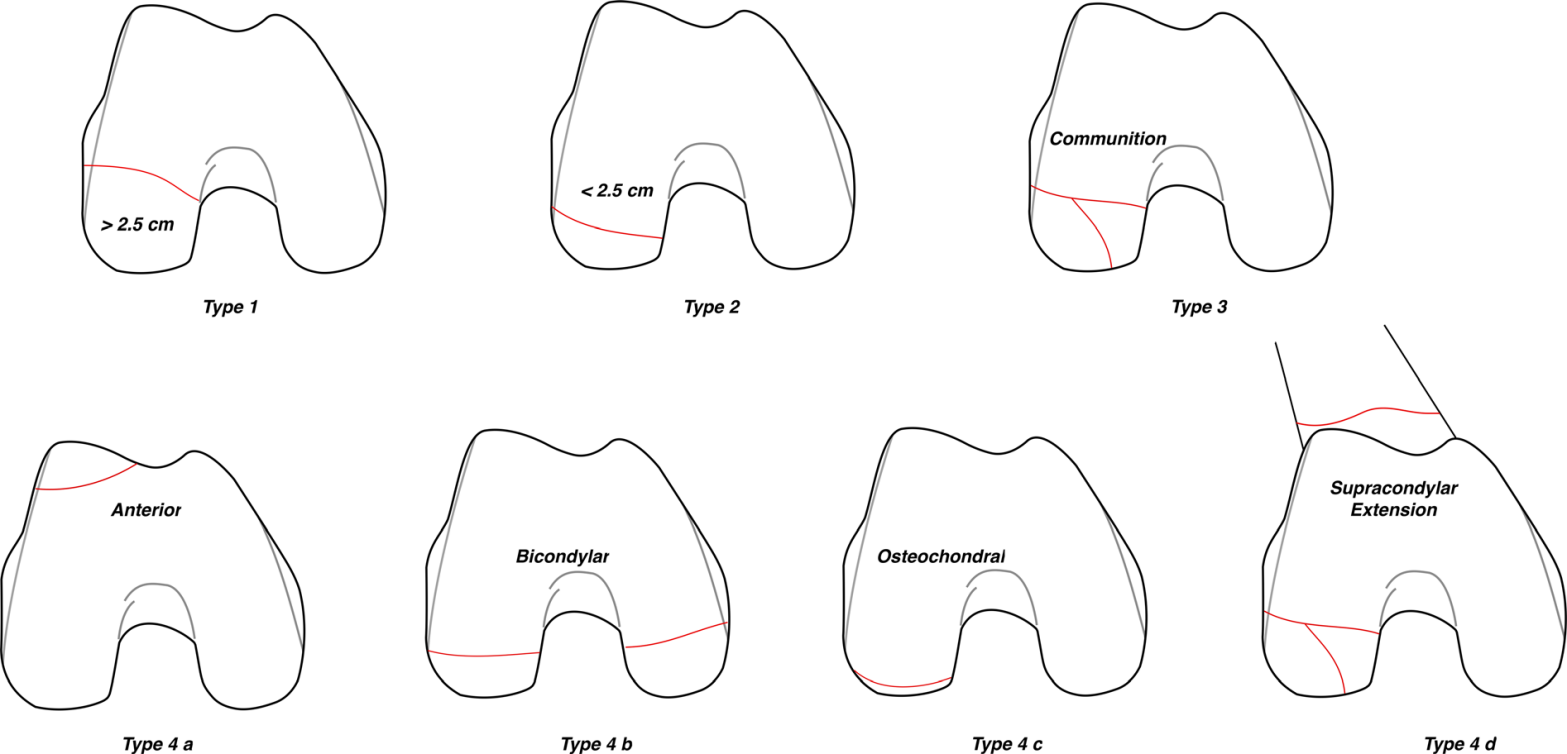
Axial View of various fracture configurations of Hoffa’s fracture as per the proposed classification system. Type 1 – unicondylar fracture with fragment size > 2.5 cm, Type 2 – unicondylar fracture with fragment size < 2.5 cm, Type 3 – comminuted fracture, Type 4a – anterior lip Hoffa’s fracture, Type 4b – bicondylar fracture, Type 4c – marginal osteochondral fracture, Type 4d – with associated supracondylar fracture.

### Inter-observer and Intra-observer variability test

Six independent surgeons of different experience levels (two consultants, two clinical assistants, and two residents) were given a set of randomly selected 15 radiographs and CT scans of Hoffa’s fracture to classify them as per the proposed classification system and to test the inter-observer reliability. This was done at two different occasions at an interval of three weeks to assess the intra-observer variability.

## Results

### A proposed classification system for Hoffa’s fracture

The classification system was primarily based on the fracture patterns seen on the CT scan images (Figures 2–4).

*Type 1* – The fracture line passes coronally at or close to the junction of posterior femoral condyle and shaft of the femur. The size of the fragment is >2.5 cm from the tip of the posterior-most point of posterior condyle.
*Type 2* – The fracture line passes posterior to the junction of posterior femoral condyle and shaft with the size of the fragment < 2.5 cm.
*Type 3* – Comminuted coronal fractures of the femoral condyle.
*Type 4* – Special types:
  1. Anterior lip Hoffa’s fracture;
  2. Bicondylar Hoffa’s fracture;
  3. Marginal osteochondral Hoffa’s fracture;
  4. Hoffa’s fractures with an associated supracondylar fracture of the distal femur.

The number of cases representing each of the fracture patterns from the proposed classification found in our series and also in the literature search is mentioned in Table 1. Unicondylar Hoffa’s (Type 1/Type 2 or medial/lateral) were the most common fractures seen in our series (53.3%) as well as in the literature (57.4%) which was followed by Hoffa’s associated with supracondylar femur fracture accounting for 102 cases (24.7%).

**Table 1:** Summary of the proposed classification for Hoffa’s fracture along with its recommended treatment modality.

Classification	Description	Proposed treatment	Approach
Type1	Unicondylar with <2.5 cm fragment size	Anterior to posterior lag screws	Standard parapatellar approach (medial or lateral)
Type 2	Unicondylar with >2.5 cm fragment size	Posterior to anterior lag screws	Swashbucker approach/Gerdy’s tubercle osteotomy for lateral condyle and subvastus approach for medial condyle
Type 3	Comminuted fracture	Lag screws augmented with a buttress plate	Swashbucker approach/Gerdy’s tubercle osteotomy for lateral condyle and subvastus approach for medial condyle
Type 4a	Anterior Hoffa’s	Anterior to posterior lag screw	Standard parapatellar approach (medial or lateral)
Type 4b	Bicondylar Hoffa’s	Lag screws depending upon the size of the fracture fragment	Swashbuckler approach or combination of medial and lateral approaches
Type 4c	Osteochondral Hoffa’s	Headless screws or bioabsorbable pins	Arthroscopic fixation
Type 4d	With associated supracondylar fracture	Lag screws with a rigid locking plate	Swashbucker approach for lateral condyle and subvastus approach for medial condyle

#### Inter-observer and intra-observer correlation

Inter and intra-observer variability for CT images demonstrated high agreement as compared to a separate correlation analysis done using X-rays. The chance-corrected and weighted kappa statistics for observer agreement, both for inter-observer and intra-observer variability demonstrated satisfactory repeatability of the classification system.

#### CT group

The %overall agreement was 0.907692 with a fixed marginal Kappa of 0.881067 and free Marginal Kappa at 0.892308. The intra-observer agreement was 1.0.

#### X-ray group

The %overall agreement was 0.73333 with a fixed marginal Kappa of 0.702431 and free-marginal Kappa at 0.735556. The intra-observer agreement was 1.0

### Proposed treatment modality based on proposed classification

*Type 1* – These fractures should be treated with an anterior to posterior lag screw as the fragment size is >2.5 cm.

*Type 2* – These fractures should be treated using the posterior to anterior lag screws due to its relatively small size.

*Type 3* – These being comminuted fractures should be stabilized using a plate fixation in buttress mode with or without lag screws.

*Type 4a* – Anterior Hoffa’s to be fixed with an anterior to posterior lag screw.

*Type 4b* – These involve both the condyles and need to be treated as per the size of the fragment and comminution. Therefore, it could be fixed with either a lag screw from anterior to posterior direction or posterior to anterior direction with or without a buttress plate or a combination of both.

*Type 4c* – These are osteochondral fragments and should be fixed with headless screws or pins.

*Type 4d* – Hoffa’s fracture involving supracondylar fractures should be treated with a rigid locking plate along with the inter-fragmentary screws.

The summary of the proposed classification system for Hoffa’s fracture is provided in Table 2.

**Table 2:** Fractures types seen in ours study group based on proposed classification system.

Type of Fracture	No. of cases (30)	Cases reported in literature (412)
Type 1 (unicondylar > 2.5 mm)	9 (30%)	Medial – 86 (20.8%)
Type 2 (unicondylar < 2.5 mm)	7 (23.3%)	Lateral – 151 (36.6%)
Type 3 (comminuted)	6 (20%)	1 (0.24%)
Type 4a (anterior)	3 (10%)	1 (0.24%)
Type 4b (bicondylar)	2 (6.67%)	26 (6.3%)
Type 4c (osteochondral)	2 (6.67%)	3 (0.72%)
Type 4d (associated supracondylar fracture)	1 (3.34%)	102 (24.7%)
Unspecified	–	43* (10.4%)

* Fracture type not mentioned.

**Table 3:** Management strategy adopted for various fracture types described in literature.

Modality used	Configuration	Cases
Screws	Anterior to posterior	202
	Posterior to anterior	28
	Screws in different configurations	15
Plates with screws	Locking compression plates/connectors with 4/6.5 mm screws	73
Plates alone	Locking compression plates/semi tubular plates	12
Conservative	–	2
Unspecified	Treatment not mentioned	80

## Discussion

Hoffa’s fractures are one of the infrequent complex knee injuries, which can sometimes be missed on plain radiographs. Hoffa’s fracture can involve either one [1, 2, 4, 5, 9] or both the condyles [9–13]. There can be a bilateral involvement [14], it can occur as a result of either open [10, 12] or closed injuries with or without communion or supracondylar extension [15]. These injuries usually are a result of very high-velocity trauma with a direct or indirect impact on a knee with more than 90° of flexion and a combination of axial loading and shearing forces [1, 4, 16]. Lateral femoral condyle coronal fractures are known to be three times more common than medial condyle fractures [1, 17]. This was also confirmed in the literature review done in this study.

The diagnosis of these fractures in the clinical practice remains to be a continuous challenge especially when they occur in isolation. These fractures are often missed on initial antero-posterior and lateral radiographs due to the coronal nature of the fracture line [4, 11, 18]. Moreover, due to the variation in the X-ray beam, the fracture line becomes obscured leading to under diagnosis of these notorious injuries especially when undisplaced. In a retrospective review by Nork et al. [15], the AP and lateral radiographs failed to diagnose more than 30% of the cases of Hoffa’s fracture. They observed that in 10.5% cases without pre-operative CT scans, these fractures were diagnosed intra-operatively. The present consensus is strongly in favour of stressing the importance of pre-operative CT scans in order to have a proper pre-operative planning.

The first prognostic classification for these complex injuries was proposed by Letenneur et al. [5] in order to predict the risk of avascular necrosis with respect to the fracture line orientation. Nevertheless, this classification was abandoned following a study by Lewis et al. [4] showing no relationship between the incidence of avascular necrosis and the fracture type. AO/OTA group tried to bring the much-needed uniformity in classifying various types of fracture happening in the entire body. Under the system, these fractures were classified as 33B3.2 for unicondylar fracture and 33B3.3 for bicondylar involvement. While this system gave identification to these fractures, it was incapable of making the distinction amongst various subtypes of these fractures that varied greatly in terms of the management approach required and their prognosis.

An ideal classification system should accomplish certain well-defined objectives which includes providing an adequate documentation of variety and subtypes of fractures, universal applicability and providing a common language for discussion among various peers. It should also be easy to understand “user-friendly” and have a role in decision making and prognostication. While all of the objectives may not be necessarily achieved in a single classification, the most important is decision making and treatment planning. The proposed working classification system can be used with X-ray, but since it has shown to have relatively poor agreement among observers compared to CT scan, it is best used in conjugation with CT, as CT is most commonly performed for planning the management of these fractures. Additionally, measuring the fragment size may not be accurate on plain radiographs.

**Figure 5:**
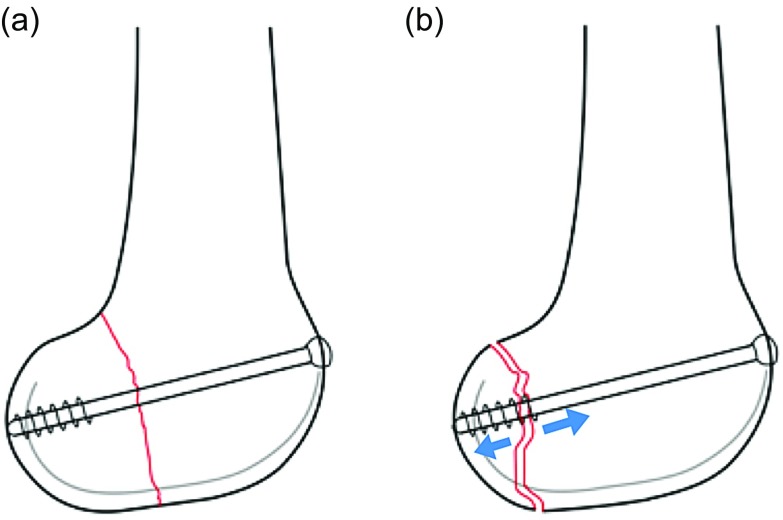
(a) Reduction of Type I Hoffa’s with AP lag screw. (b) Improper reduction of Type II Hoffa’s with AP lag screw leading to distraction at the fracture site (indicated by blue arrows).

In the proposed classification system of Hoffa’s fracture, “*Type 1*” indicates unicondylar fracture with fragment size > 2.5 cm and should be treated using lag screws in anterior to posterior direction using standard medial or lateral parapatellar approach. The fragment size of 2.5 cm was chosen by considering a 50% error margin for a 16 mm partially threaded screw grip strength (16 × 1.5 = 24 mm, rounded off to 25 mm). The principle of the lag screw is based on the premise that the threads will engage in the distal fragment allowing it to be pulled to the proximal or main fragment producing compression at the fracture site (Figure 5a).

“*Type 2*” fractures are the one where the fracture fragment is smaller than 2.5 cm. Owing to the smaller size, the lag screws inserted in the anterior to posterior direction might give an inadequate compression and have insufficient hold in the distal fragment and can even lead to distraction at the fracture site (Figure 5b). Therefore, we propose that a screw inserted in posterior to anterior direction would be appropriate. For this either swashbuckler approach [19, 20]/Gerdy’s tubercle osteotomy for lateral condyle [21] and subvastus approach for medial condyle would provide a better exposure [9]. We found that anterior to posterior lag screw was the most common method used for treating Hoffa’s fracture in the literature even when the fragment is small, even though postero-anterior screws have shown to have more biomechanical stability [22] (Table 3). This could be mainly due to the simplicity of the technique and the reasonable stability offered.

“*Type 3*” fractures are the ones with comminution. These fractures are intrinsically unstable owing to the presence of shear forces in flexion and extension and thus there is a necessity to apply an additional buttress plate to improve the fracture stability [23]. While a good reduction in non-comminuted Type I and II fracture with good inter-digitation of fracture fragments ensures primary stability, in the presence of comminution this is non-existent making lag screw alone vulnerable to fail in face of the deforming forces. A plate in buttress mode provides resistance to shear forces across the fracture. Again, Swashbuckler approach/Gerdy’s tubercle osteotomy or subvastus approach should be used for better visibility of posterior condyle articular surface and comminution [14, 19–21].

“*Type 4*” fractures constitute special types of coronal fractures that need to be managed individually on their own merit. “*Type 4a*” is an anterior lip Hoffa’s fracture which is rare and is traditionally classified as 33B3.1 by AO/OTA classification [8]. However, it belongs to a similar category of coronal split or shear fractures and management would include putting anteroposterior lag screws avoiding the articulating surfaces. Albeit less common, this type has been included first for the sake of simplicity (“a” for anterior). Being a mirror replica of Type II fractures, they can be treated successfully using a lag screw in anterior to posterior direction for adequate compression using standard parapatellar approach.

“*Type 4b*” is the one with bicondylar involvement (“b” for bicondylar). These have been classified by AO/OTA classification as 33B3.3 [8]. These fractures can be treated as two different entities depending on the fracture geometry, fragment size, and comminution. For instance, if the fragment is >2.5 cm, then an anterior to posterior screw can suffice while a posterior to anterior screw is essential for a smaller fragment. Double incision or Swashbuckler approach can be used [19].

“*Type 4c*” are marginal osteochondral fractures (“c” for cartilage) which are predominantly cartilaginous or at best tiny shell of an osteocartilaginous fragment that can be best managed by Open or arthroscopic reduction using bioabsorbable wires/headless screws depending upon the condyle involved [24, 25].

“*Type 4d*” is a Hoffa’s fracture with associated supracondylar fractures of the distal femur. This association has been seen in up to 38% cases, which can alter the management plan for distal femur fractures [20]. Use of longer, more rigid fixation with a locking buttress plate construct augmented by lag screws has been recommended [15]. Swashbuckler or subvastus approach is preferred. The diagnosis of Hoffa’s fractures involves a multimodal approach which includes clinical and radiological evaluation. As these are coronal plane fractures, there are high chances of missing the fracture, especially if associated with supracondylar–intercondylar extension and so pre-operative computerized tomography is strongly recommended [20]. Additionally, CT scan provides clear anatomic details, true fracture size, and aids in planning the surgical approach and optimum fixation strategy.

Retrospective nature remains the primary limitation of the present study, however, majority of the classifications systems are based on retrospective case studies, surgeon’s experience and understanding of the fracture geometry. This classification study may also be termed as arbitrary as it is not based on any biomechanical or long-term follow-up prognostication analysis. The observational study involves our own cases and the ones reported in literature covering the entire spectrum of these injuries, which despite being different in their character and management approach required were hitherto clubbed in one group of fracture under a single group.

To the best of our knowledge, this is the first study of its kind which describes a CT and radiograph based classification for this infrequent injury pattern along with the rationale for its management. A thorough review of the literature about various patterns of Hoffa’s fracture was done which is the strength of the present study. The fact that there was substantial concurrence in their classification and agreement on the management of these cases suggest that the classification system has several practical advantages in a clinical scenario.

In conclusion, the proposed CT based classification of Hoffa’s fracture can give an insight in terms of better pre-operative planning and can also help in formulating the treatment plan. It can also help in determining the prognosis for these rare injuries in a better way. The intra and inter-observer variability tests prove good reproducibility for this classification.

## References

[1] Manfredini M, Gildone A, Ferrante R, Bernasconi S, Massari L (2001) Unicondylar femoral fractures: A review of 23 patients. Acta Orthop Belgia 6(2), 132–138. 11383291

[2] Arastu MH, Kokke MC, Duffy PJ, Korley RE, Buckley RE (2013) Coronal plane partial articular fractures of the distal femoral condyle: Current concepts in management. Bone Joint J 95-B(9), 1165–1171. 23997126 10.1302/0301-620X.95B9.30656

[3] Bartoníček J, Rammelt S (2015) History of femoral head fracture and coronal fracture of the femoral condyles. Int Orthop 39(6), 1245–1250. 25787681 10.1007/s00264-015-2730-x

[4] Lewis SL, Pozo JL, Muirhead Allwood WF (1989) Coronal fractures of the lateral femoral condyle. J Bone Joint Surg Br 71(1), 118–120. 2914979 10.1302/0301-620X.71B1.2914979

[5] Letenneur J, Labour PE, Rogez JM, Lignon J, Bainvel JV (1978) Fractures de Hoffa: a propos de 20 observations. Ann Chir 32(3–4), 213–219. 697301

[6] Ostermann PA, Neumann K, Ekkernkamp A, Muhr G (1994) Long term results of unicondylar fractures of the femur. J Orthop Trauma 8(2), 142–146. 8207571 10.1097/00005131-199404000-00011

[7] Shi J, Tao J, Zhou Z, Gao M (2014) Surgical treatment of lateral Hoffa fracture with a locking plate through the lateral approach. Eur J Orthop Surg Traumatol 24(4), 587–592. 23615680 10.1007/s00590-013-1224-z

[8] Kellam JF, Meinberg EG, Agel J, Karam MD, Roberts CS (2018) Introduction: Fracture and dislocation classification compendium – 2018: International Comprehensive Classification of Fractures and Dislocations Committee. J Ortho Trauma 32(Suppl 1), S1–S10. 10.1097/BOT.0000000000001063 29256945

[9] Viskontas DG, Nork SE, Barei DP, Dunbar R (2010) Technique of reduction and fixation of unicondylar medial Hoffa fracture. Am J Orthop (Belle Mead NJ) 39(9), 424–428. 21290019

[10] Calmet J, Mellado JM, García Forcada IL, Giné J (2004) Open bicondylar Hoffa fracture associated with extensor mechanism injury. J Orthop Trauma 18(5), 323–325. 15105758 10.1097/00005131-200405000-00012

[11] Papadopoulos AX, Panagopoulos A, Karageorgos A, Tyllianakis M (2004) Operative treatment of unilateral bicondylar Hoffa fractures. J Orthop Trauma 18(2), 119–122. 14743034 10.1097/00005131-200402000-00012

[12] Kini SG, Sharma M, Raman R (2013) A rare case of open bicondylar Hoffa fracture with extensor mechanism disruption. BMJ Case Rep 2013, bcr2013009541. 10.1136/bcr-2013-009541 PMC3669886 23645653

[13] Mounasamy V, Hickerson L, Fehring K, Desai P (2013) Open Bicondylar Hoffa fracture with patella fracture: A case report and literature review. Eur J Orthop Surg Traumatol 23(Suppl 2), S261–S265. 23443745 10.1007/s00590-013-1194-1

[14] Koné S, Bana A, Touré SA, Koné S, Allou A-S, Kouassi AN, Koffi AG, Kouamé IM (2015) Hoffa fracture of medial unicondylar and bilateral in a man: A rare case. Pan Afr Med J 20, 382. 26185572 10.11604/pamj.2015.20.382.6092 PMC4499274

[15] Nork SE, Segina DN, Aflatoon K, Barei DP, Henley MB, Holt S, Benirschke SK (2005) The association between supracondylar-intercondylar distal femoral fractures and coronal plane fractures. J Bone Joint Surg Am 87(3), 564–569. 15741623 10.2106/JBJS.D.01751

[16] Dhillon MS, Mootha AK, Bali K, Prabhakar S, Dhatt SS, Kumar V (2012) Coronal fractures of the medial femoral condyle: A series of 6 cases and review of literature. Musculoskelet Surg 96, 49–54. 21904943 10.1007/s12306-011-0165-0

[17] Martelli S, Pinskerova V (2002) The shapes of the tibial and femoral articular surfaces in relation to tibiofemoral movement. J Bone Joint Surg [Br] 84-B, 607–613. 10.1302/0301-620x.84b4.12149 12043788

[18] Holmes SM, Bomback D, Baumgaertner MR (2004) Coronal fractures of the femoral condyle: A brief report of five cases. J Orthop Trauma 18(5), 316–319. 15105756 10.1097/00005131-200405000-00010

[19] Dua A, Shamshery PK (2010) Bicondylar Hoffa fracture: Open reduction internal fixation using the swashbuckler approach. J Knee Surg 23(1), 21–24. 20812577 10.1055/s-0030-1262319

[20] Sharath RK, Gadi D, Grover A, Gour SK (2015) Operative treatment of isolated bicondylar hoffa fracture with a modified swashbuckler approach. Arch Trauma Res 4(4), e25313. 26848468 10.5812/atr.25313 PMC4733513

[21] Liebergall M, Wilber H, Mosheiff R, Segal D (2000) Gerdy’s tubercle osteotomy for the treatment of coronal fractures of the lateral femoral condyle. J Orthop Trauma 14(3), 214–215. 10791675 10.1097/00005131-200003000-00013

[22] Jarit GJ, Kummer FJ, Gibber MJ, Egol KA (2006) A mechanical evaluation of two fixation methods using cancelloous screws for doronal fractures of lateral femoral condyle of the distal femur (OTA type 33B). J Orthop Trauma 20(4), 273–276. 16721243 10.1097/00005131-200604000-00007

[23] Cheng PL, Choi SH, Hsu YC (2009) Hoffa fracture: Should precautions be taken during fixation and rehabilitation? Hong Kong Med J 15(5), 385–387. 19801698

[24] Borse V, Hahnel J, Cohen A (2010) Hoffa fracture: Fixation using headless compression screws. Eur J Trauma Emerg Surg 36(5), 477–479. 26816229 10.1007/s00068-010-0014-0

[25] Kapoor C, Merh A, Shah M, Golwala P (2016) A case of distal femur medial condyle hoffa type II(C) fracture treated with headless screws. Cureus 8(9), e802. 27790391 10.7759/cureus.802 PMC5081261

